# Parents' knowledge of their child with profound intellectual and multiple disabilities: An interpretative synthesis

**DOI:** 10.1111/jar.12740

**Published:** 2020-05-05

**Authors:** Kasper Kruithof, Dick Willems, Faridi van Etten‐Jamaludin, Erik Olsman

**Affiliations:** ^1^ Section of Medical Ethics Department of General Practice Amsterdam UMC Location AMC University of Amsterdam Amsterdam The Netherlands; ^2^ Department of Medical Ethics & Health Law Leiden University Medical Center Leiden The Netherlands; ^3^ Department of Spiritual Care Hoofddorp The Netherlands

**Keywords:** ageing, disability, knowledge, parents, PIMD

## Abstract

**Background:**

Parents’ knowledge of their child with profound intellectual and multiple disabilities (PIMD) is seen as crucial in the support and care for their child. The aim of this study was to explore the nature and transferability of this knowledge.

**Method:**

We conducted an interpretative synthesis, searched PubMed, CINAHL, Philosopher's Index and PsycINFO and included fourteen studies.

**Results:**

Parents’ knowledge was based on their long‐lasting and special bond with their child and described as an intuition, a gut feeling, a sixth sense and a sense of knowing. Parents applied their knowledge as experts in interpreting their child's intended communication, well‐being and pain, and as advocates opposing the more objectivist approach of medical professionals. Showing by example and passing on narratives were seen as important ways of transferring this knowledge.

**Conclusions:**

Suggestions are made on how to apply and retain parents’ knowledge to improve care and support for people with PIMD.

## 1 INTRODUCTION

Persons with profound intellectual and multiple disabilities (PIMD) have an estimated IQ below 20. They have profound neuromotor dysfunctions, often accompanied by sensory impairments and medical problems, such as seizures, respiratory and feeding problems (Nakken & Vlaskamp, 2007). Persons with PIMD have little or no understanding of verbal language and no apparent symbolic interaction with objects and are therefore always dependent on others (Nakken & Vlaskamp, 2007). This dependency means that parents of persons with PIMD play a large, often lifelong (Seltzer, Greenberg, Floyd, Pettee, & Hong, 2001), role in the lives of their child. Although most people with PIMD live in professional care settings, some of them, in all age groups, live at home. Rough estimations in the Netherlands suggest percentages of between 2.4% and 13% of people with PIMD being cared for in their family home (Schuurman, 2010; Vlaskamp, 2002; Vugteveen, Putten, & Vlaskamp, 2014). However, the ambiguous description of the group in literature and surveys makes that especially the numbers of people with PIMD living at home may be underestimated (Vugteveen et al., 2014).

The literature suggests that parents have unique and crucial knowledge of their child with PIMD, which helps them, for example, to improve the support and care of the child, regardless of whether the child is living at home or not (Gauthier‐Boudreault, Gallagher, & Couture, 2017; Jokinen & Brown, 2005). For example, if persons with PIMD live at a residential care facility or go to a day activity centre, parents are often involved in personalizing the care for their child, monitoring this care and making decisions on their behalf (de Geeter, Poppes, & Vlaskamp, 2002; Jansen, Putten, & Vlaskamp, 2013). In addition, parents’ knowledge of their child with PIMD can be seen as a main driver in the continuity of support and care for people with PIMD (Kamstra, Van der Putten, & Vlaskamp, 2015), especially because the turnover of personnel in professional care organizations is often high (Stringer, Terry, Ryan, & Pike, 2018) and care and support for people with PIMD involves a large number of professional caregivers. Finally, if persons with PIMD live at home, it could be that a day will come that parents cannot provide the support and care for their child anymore, and that this support and care has to be transferred to others. Since life expectancy of persons with PIMD has been growing, and the chance that they outlive their parents is increasing (Jokinen & Brown, 2005; Braddock, Hemp, & Rizzolo, 2004), this transfer of support and care can be sudden and/or involuntary as well. Moreover, if parents are outlived by their child with PIMD, this may to some extent result in the loss of certain knowledge about their child.

Since parents’ knowledge of their child with PIMD seems crucial in the support and care for people with PIMD, we need a better understanding of this knowledge. When we know more about the nature of parents’ knowledge and how they apply it, we can address its implications for the care and support for people with PIMD. Furthermore, in doing this we aim to explore the transferability of parents’ knowledge to others, which can be crucial in retaining high quality care and support for people with PIMD. This transferability could be especially important within the context of an increased likelihood of parents being outlived by their child with PIMD. To the best of our knowledge, the literature on parents’ knowledge of their child with PIMD has not yet been explored systematically.

### 1.1 Aim & research questions

The objective of this study was to explore parents’ knowledge of their child with PIMD. We did this by trying to answer the following research questions: (1) What is the nature of parents’ knowledge of their child with PIMD? (2) How do they use this knowledge? (3) Is this knowledge transferable to others?

## 2 METHOD

### 2.1 Design

We conducted a narrative interpretative synthesis (Athanasiou & Darzi, 2011; Dixon‐Woods, Agarwal, Jones, Young, & Sutton, 2005) about the particular knowledge that parents have about their child with PIMD. This means that we not only selected and categorized what was known, that is made an integrative synthesis, but also tried to relate this knowledge to theoretical concepts, that is developing a theory based on what is known.

### 2.2 Literature search

**Figure 1:**
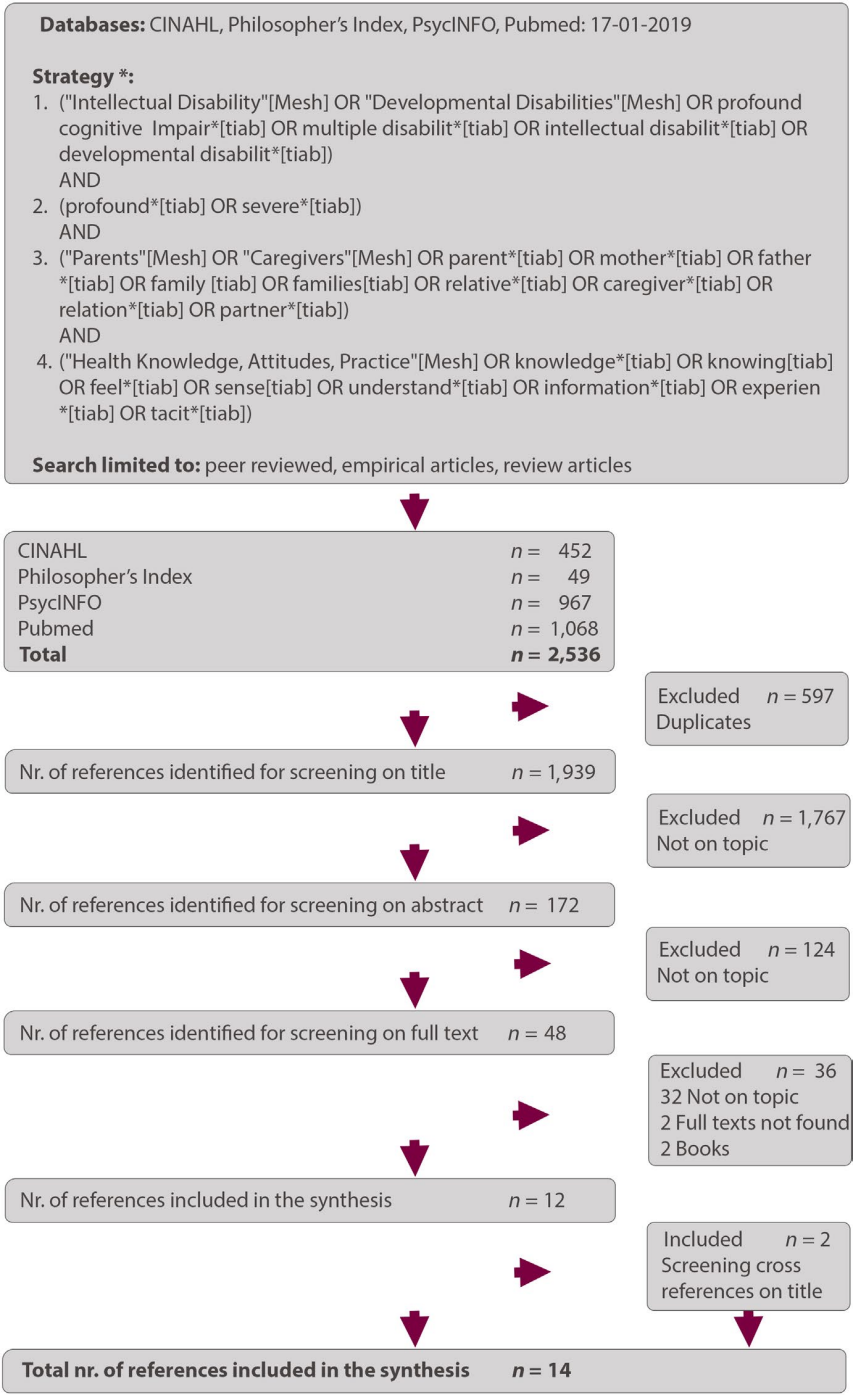
Flow chart. * = Flowchart based on the search strategy in PubMed, see supplementary file 1 for details on the searches in the other databases

We searched for references in PubMed, CINAHL, Philosophers’ Index and PsycINFO in January 2019. We developed a search strategy with the help of a professional clinical librarian. This led to the following searches (see Figure 1): (a) intellectual disabilit*—or synonyms/ alternatives as a mesh term or in the title or abstract. (b) *severe* or profound* in the title or abstract. (c) parent*—or synonyms/ alternatives—as mesh term or in the title or abstract. (d) knowledge*—or synonyms/ alternatives—as mesh term or in the title or abstract. These four searches were combined with the Boolean operator AND (see Figure 1). For an overview of the complete search strategy, see Appendix S1.

We refined the search strategy several times, but this did not alter our initial search strategy. For instance, since the terminology about people with disabilities has changed over time, we added the option OR handicap* (title/abstract) in our PubMed search in search 1. This resulted in 243 additional hits, only one of which was selected on the basis of its title, yet was excluded after reading the full text. This made us decide not to adjust our search strategy for the other databases, since the yield was too meagre. In addition, we wanted to find out what we had missed through possible ambiguities in the description of our group of interest in the existing literature. To this end, we eliminated search 2 (*severe* or profound*) entirely, which resulted in 7,226 hits in total. After screening the first 200 titles arranged by date from new to old and then screening the first 200 titles arranged from old to new, we found no additional references that could be included. As a consequence, we did not adjust our search strategy to be more inclusive.

### 2.3 Procedure

Since there were not many studies about the topic of experiential knowledge of parents of children with PIMD, we included all studies that referred to parents’ knowledge of persons with PIMD, even when this was not the main topic of the article. KK screened the titles and abstracts, based on the following inclusion criteria:

Describing parents' knowledge of their child with severe or profound disabilities
Empirical study or review study based on empirical research
Published in a peer‐reviewed journal
Published in English, German, French, Spanish or Dutch

In case of doubt, KK included the studies for the next round. KK then screened the full texts, and in case of doubt, he asked EO to screen the full text independently, after which they discussed it until consensus was reached.

### 2.4 Data evaluation

The final sample for this interpretative synthesis included mostly qualitative studies, but also some literature reviews and quantitative studies. Various methods, such as in‐depth interviews, text analyses and questionnaires, were used. The included studies were critically appraised regarding both study design and data analysis, while for the qualitative studies, we used the COREQ checklist (Tong, Sainsbury, & Craig, 2007). For the quantitative studies, we used a tool developed by Jack et al. (2010). We described a risk of bias for the included studies. None of the retrieved articles were excluded by this critical appraisal.

### 2.5 Data extraction and analysis

The characteristics of the included studies were described, which resulted in Table 1. We then marked segments of the full texts that mentioned the knowledge of parents regarding the person with PIMD. The first author attached thematic codes to these segments (Miles, Huberman, & Saldana, 2014). Segments were coded based on our research questions, as: (a) the nature of parents’ knowledge, (b) the roles parents fulfil with their knowledge, and (c) ways of exchanging this knowledge with professionals. The first, second and last authors discussed the coded segments. Consensus was reached on the included segments. To check whether any segments could have been missed, the last author independently coded segments in three articles. This did not result in changes. For the thematic analysis, see Appendix S2.

**Table 1:** Characteristics of the included articles

1st author	Year	Objective	Study design	Participants	Country	Risk of Bias
Axelsson	2014	To identify ways to facilitate participation in family activities for children and adolescents with profound intellectual and multiple disabilities (PIMD).	Qualitative: interviews.	Parents (*n* = 11) Professionals (*n* = 9)	Sweden	Interviews were conducted by telephone
Carter	2002	To explore the ways in which parents of children with profound special needs assess and manage their children's pain.	Qualitative: semi‐structured interviews.	Parents (*n* = 15)	The United Kingdom	Date of the study
Carter	2017	To explore how parents acquire knowledge and skills to assess and manage their child's pain.	Mixed methods: pain survey and interviews.	Parents (*n* = 8)	The United Kingdom	Small sample. Aim and sample size suggest qualitative approach
Fonteine	2007	To explore the effectiveness and efficiency of communication logs.	Qualitative: text analysis.	Parents (*n* = 12)	The Netherlands	‐
Gauthier‐Boudreault	2017	To propose realistic solutions to meet the needs of young adults with profound intellectual disability and their families during and after the transition to adulthood.	Qualitative: semi‐structured interviews.	Parents (*n* = 14)	Canada	Snowball sampling was used^a^
Gauthier‐Boudreault	2017	To document the needs of parents and young adults with profound intellectual disability during and after the transition to adulthood.	Qualitative: semi‐structured interviews.	Parents (*n* = 14)	Canada	Snowball sampling was used^a^
Geeter	2002	To demonstrate the supposition that co‐operation between parents and professionals must meet certain criteria if parents are to receive a proper chance of using their existing knowledge, while at the same time adding to their skills.	Quantitative: Questionnaire.	Parents (*n* = 723)	The Netherlands	Date of the study
Graham	2009	To describe the experience of paediatric intensive care hospitalization from the perspective of parents of children with severe, antecedent disability.	Qualitative: semi‐structured interviews.	Parents (*n* = 8)	The United States of America	Small sample. Poor description of characteristics of the children
Hostyn	2009	To describe and synthesize studies addressing the interaction between persons with PIMD and their partners.	Literature review.	NA	Belgium	‐
Hunt	2002	To explore the diagnostic and clinical decision‐making processes used by parents and healthcare professionals in relation to pain in children with severe to profound neurological impairment.	Qualitative: semi‐structured interviews.	Parents (*n* = 20) Professionals (*n* = 26)	The United Kingdom	Date of the study
Stringer	2018	To understand caregivers’ perspectives on the development of the patient–physician relationship for adult patients with severe or profound intellectual developmental disabilities.	Qualitative: semi‐structured interviews.	Parents (*n* = 6) Other family members (*n* = 4) Professionals (*n* = 3)	Canada	Presented quotations do not Distinguish between parents, other family members and professionals. Poor description of data analysis
Watson	2017	To characterize supported decision making for people with severe or profound intellectual disability.	Qualitative: Observations and informal talks.	Unpaid primary caregivers (*n* = 8) Professionals (*n* = 25)	Australia	Presented quotations do not distinguish between parents and other unpaid primary caregivers^b^
Zaal‐Schuller	2016	To understand how parents of children with severe developmental disorders experience their involvement in end‐of‐life decision making.	Literature review.	NA	The Netherlands	‐
Zaal‐Schuller	2016	To investigate the experiences of the parents and the physician involved during the end‐of‐life decision‐making (EoLDM) process for children with PIMD.	Qualitative: semi‐structured interviews.	Parents (*n* = 17) Professionals (*n* = 11)	The Netherlands	Limited description of data analysis

Abbreviation: NA, Not applicable.

^a^ These two studies used the same dataset.

^b^ In the Methods section, it was not specified who the unpaid primary caregivers were. At least some of them were explicitly presented as mothers in the results section.

## 3 RESULTS

Fourteen articles were included. For the characteristics of the included articles, see Table 1. The thematic analysis led to four main themes: the nature of parents’ knowledge, parents as experts, parents as advocates and transfer of knowledge.

### 3.1 The nature of parents' knowledge

Parents’ knowledge was based on their special bond with their child, which they had developed through proximity and over time (de Geeter et al., 2002). The knowledge was described as a “sense of knowing,” a “sixth sense” or a “gut feeling” which developed because parents “were attuned to their child through being a constant presence” (Carter, Arnott, Simons, & Bray, 2017, p. 9). They could “read” their child and “felt guided by a sense of their child's condition and needs” (Zaal‐Schuller, de Vos, Ewals, van Goudoever, & Willems, 2016) or had knowledge of their child in an intuitive way, which differed from the knowledge of medical professionals (Carter et al., 2017; de Geeter et al., 2002).

### 3.2 The parent as expert

Parents saw themselves, based on their knowledge, as being best equipped to interpret their child's attempts to communicate (Stringer et al., 2018). According to the parents, professional caregivers and medical professionals in several studies, parents were the experts on their child and applied their expert knowledge in various fields, such as their child's well‐being or the lack thereof (Axelsson, Imms, & Wilder, 2014; Carter et al., 2017; Hostyn & Maes, 2009; Zaal‐Schuller, Willems, Ewals, van Goudoever, & de Vos, 2016; de Geeter et al., 2002). For example, parents in a qualitative study fulfilled the role of expert during end‐of‐life decision‐making (EoLDM) processes by “reading” their child; they could “feel” that their child was deteriorating before the physician became aware of it (Zaal‐Schuller, Willems, et al., 2016).

Parents were also seen, by both themselves and professionals, as experts when it came to understanding their child's pain. Hunt, Mastroyannopoulou, Goldman, and Seers (2003, p. 177) used semi‐structured interviews with parents and professionals to show that parents “just knew” when their child was in pain, which was “an intuition,” “a feeling,” “something that comes from within.” The findings by Carter, McArthur, and Cunliffe (2002), based on pain diaries and interviews, showed something similar. Their study reported that professionals felt uncertain and lacked confidence regarding the pain assessment of persons with PIMD, and their knowledge was more fragmented than that of the parents. They therefore often turned to the parents, especially the mothers, who had developed a “sense of knowing” through trial and error, and were seen as more able to “join up the dots” (Carter et al., 2017, p. 7).

Others stated that the knowledge of parents, being the experts about their child, should be recognized in care work in order to improve care (Gauthier‐Boudreault et al., 2017; de Geeter et al., 2002). For example, de Geeter et al. (2002) argued that professional caregivers should create space for the experiential knowledge of parents. Using a questionnaire (*n* = 723), they showed how co‐creation between parents and professional caregivers resulted in parents being more satisfied with the care for their child.

### 3.3 The parent as advocate

Parents also utilized their knowledge of their child with PIMD in their role as advocates, counteracting the more objectivist approach of medical professionals (Carter et al., 2017; Graham, Pemstein, & Curley, 2009; Zaal‐Schuller, Willems, et al., 2016), for instance when the latter had no personal knowledge of their child (Gauthier‐Boudreault, Couture, & Gallagher, 2018). In their review of the literature on end‐of‐life decisions, Zaal‐Schuller, Willems, et al. (2016) showed how parents took on the role of advocates. As long as parents could still “sense” their child's conditions and needs, and saw the child's life as “positive and enriching,” they strongly advocated for life‐prolonging measures (Zaal‐Schuller, Vos, et al., 2016, p. 243).

Using the knowledge of their child for the purpose of advocacy required that parents had the competence to “translate” their experiential knowledge into forms of objective knowledge that would be more readily accepted by medical professionals. For example, Carter et al. (2017, p. 7) showed that mothers “found it difficult to deliver the level of “proof” they thought that health professionals wanted.” Parents in another qualitative study, conducted in the United States, mentioned that they felt healthcare professionals underappreciated the level of their child's functioning, neither recognizing the parental knowledge nor the child's full potential. These parents tried to influence the views of the professionals by making a case for their child, for instance by showing pictures of happy moments when their children were at home (Graham et al., 2009).

Another study found that when physicians respected the strong bonds between parent(s) and child, including parents’ protection of their child, this could result in parents “opening up” their bond to the physician. In that case, mutual trust developed between parents and physicians, resulting in a greater appreciation by parents of the medical care for their child (Stringer et al., 2018).

### 3.4 Transfer of knowledge

Based on the findings about the nature of parents’ knowledge and the way they use this knowledge, we looked at the transferability of their knowledge. Fonteine, Zijlstra, and Vlaskamp (2008) conducted a text analysis of 12 logs used in a day activity centre for people with PIMD, focusing on the exchange of information between parents and professional caregivers. They found that parents’ knowledge was not readily transferable through a log. They argued that the rich and in‐depth experiential knowledge that parents possess should be transferred by other methods, preferably by doing and interacting. They recommended that parents should be allowed to spend some days with their child at the activity centre to show how they did things like eating or conflict‐solving, thereby transferring their “embodied knowledge.” Findings by Hunt et al. (2003) underscored the validity of these recommendations. Based on interviews with both parents and professionals, they showed how nurses got to know the patient and his/her signals by observing the ways in which parents interacted with their child.

Watson, Wilson, and Hagiliassis (2017) argued that the in‐depth, experience‐driven knowledge of parents can be indirectly transferred to others through the sharing of life stories and visual images of the person with PIMD. Based on observations and informal talks with parents and professional caregivers, they showed how these professional caregivers felt more connected to, and hence felt more able to take care of, a person with PIMD when they knew the person's stories and history. At the same time, these caregivers felt more detached from persons with PIMD than the parents and therefore found it more difficult to estimate the meaning of their interaction if they lacked knowledge about the person. Watson et al. (2017) showed that this knowledge does not necessarily have to be acquired over a lengthy period of time and through intensive interaction, but that the process can be at least accelerated through hearing stories and seeing visual images of the person with PIMD, which reflect that person's history.

## 4 DISCUSSION

### 4.1 Summary of findings

In this synthesis of empirical studies and review studies based on empirical research, we explored the nature of parents’ knowledge of their child with PIMD, the way they used this knowledge, and the transferability of this knowledge to others. The findings of our study suggest that parents possess unique, experiential knowledge of their child with PIMD, which they have gathered through intensive interactions with their child. The findings also suggest that they use their knowledge roughly in two ways: as experts they have experiential knowledge of their child's communication, well‐being and pain, and as advocates they use their knowledge to counteract the objectivism of medical approaches, or translate their knowledge into forms of knowledge that are more accepted within health care and medicine. Finally, our study indicates that the in‐depth parental knowledge of their child with PIMD might be partly transferable to professional caregivers by giving parents a role in the care for their child, so they can demonstrate their knowledge to other caregivers “by example.” Moreover, our findings suggest that when parents share life stories and images of their child with professional caregivers, this may result in these caregivers acquiring a deeper understanding of the person with PIMD.

### 4.2 Strengths and Limitations

One limitation of our review is that all included studies were conducted in Western countries, limiting the applicability of our findings to other parts of the world. Being a parent of a child with PIMD may be different in areas other than Western countries. This was described by Manaka, Wath, and Moagi (2018), who addressed the lack of parental involvement in the care for people with PIMD in South Africa. How parental involvement in the care for people with PIMD differs worldwide is a worthwhile topic for further research.

Most of the literature we found dealt with the role of parents in relation to medical professionals, while it is known that parents also often discuss the care for their child with professional support workers (Fonteine et al., 2008; de Geeter et al., 2002; Jansen et al., 2013). One possible explanation for the finding that parents’ knowledge has been more commonly studied in relation to medical decisions is that their knowledge differs most from that of medical professionals. This difference is important to address because a lot may be at stake in these situations, like decisions concerning life and death. Future research may scrutinize the relation between parents’ knowledge, the knowledge of medical professionals and that of other professionals.

Our results were based on literature published in peer‐reviewed journals. Some of them had small samples or other methodological limitations, which we addressed in Table 1. This could mean that our findings could be biased. However, the overall pattern we identified by comparing the literature was consistent. Moreover, we interpreted our findings based on related literature. Hence, we do regard our findings as relatively robust.

The systematic approach to finding out what is known regarding the parental knowledge of children with PIMD is a strength of our study. This can be seen as a first step in working towards a theoretical framework, based on empirical research. Another strength of an interpretative synthesis is its contribution to theory, and theory development is necessary as a background for future studies. Such a framework may help to overcome the challenges ahead, which are prompted by the current trend towards longer survival among the population.

### 4.3 Interpretation

The parents’ knowledge was described as a “sense of knowing” through “continuous presence”; they “read” their child, making use of their “gut feelings” and a “sixth sense” (Carter et al., 2017; Zaal‐Schuller, Willems, et al., 2016). These findings can be understood as parents describing a relation between their knowledge and their body, which is known as embodiment. This is in line with Reinders (2010), who used Polanyi's concept of “tacit knowledge” (Polanyi, 2009), in which experiential knowledge is seen as embodied knowledge. Reinders argues that, instead of trying to fit knowledge into a predetermined mould, caregivers should reach a form of “Verstehen” (Weber, 1947) of the situation of others by making use of the rich experiential knowledge that people embody in themselves through years of interaction and reflection.

Reinders (2010) emphasizes that personal embodied knowledge is paramount because it allows the possessor of this knowledge to make distinctions in the behaviour of and communication with the person with PIMD. Olsman, Nieuwenhuijse, and Willems (submitted) made a similar point when they argued that the experiential knowledge of parents can be of benefit when assessing the quality of life (QoL) of persons with PIMD. They question the usage of standardized instruments to measure the QoL of people with PIMD because this implies an objectivity that does not exist when it comes to this group, and leaves no space for the complex experiential knowledge, based on years of experiences, of the parents of a child with PIMD.

If the parents’ experiential knowledge of their child with PIMD should indeed be understood as tacit knowledge, this has implications for the transferability of this knowledge. Reinders (2010, p. 31) addressed this point explicitly when he stated that “because of its personal dimension, tacit knowledge is not readily transferable.” In other words, the knower knows what he knows of the subject because of the relationship he has with the subject and this relationship is not transferable. However, Reinders (2010) was describing the tacit knowledge of professional caregivers. Like others (Hostyn & Maes, 2009; Watson et al., 2017; Carter, Simons, Bray, & Arnott, 2016; Donovan, 2002), he classified this type of knowledge based on the emotional involvement and closeness that develops between people over years of interaction, rather than on a supposed unique bond between parent and child. Thus, the parents’ knowledge might not be readily transferable, but is acquirable by others than the parents (Carter et al., 2016; Donovan, 2002).

Interestingly, our findings indicate that the embodied or tacit knowledge possessed by parents might nevertheless be transferable, albeit partly and indirectly. In other words, others can acquire this knowledge with the help of the parents. Hunt et al. (2003) and Fonteine et al. (2008) argue that the embodied or tacit knowledge possessed by parents could be transferred to professionals through showing by example. By looking at the interaction between parents and child, in which parents “act out” their embodied knowledge, professionals can “learn” to get to know the patient and his/her particularities (Hunt et al., 2003). This could be seen as an interactionist approach to passing on knowledge (Berger & Luckmann, 1966 [reprint 1996]): parents can “show” their relationship with their child and the professional can learn from looking at this interaction. The professional can be “habitualized” (Berger & Luckmann, 1966, p. 74 [reprint 1996]) by the parents as they show by their example what works and what does not work.

Watson et al. (2017) added that parents can also transfer their tacit knowledge indirectly, by sharing life stories and the history of their child with other caregivers. This transferable knowledge helps the caregiver to better understand the person with PIMD and empathize with them (Watson et al., 2017; Charon, 2001; Keen, 2006). This understanding and empathy may in turn result in improved interaction, care and decision making (Watson et al., 2017; Charon, 2001). This can be seen as parents “enhancing” the relationship between professional caregivers and their child.

Another key finding was that parents used their knowledge roughly in two ways: as experts and as advocates. we may ask critical questions about these roles and the relationship between them. Viewing the parent as an advocate, we may ask what information parents select when they pass on their narratives, and with what intent (Graham et al., 2009). In other words, how do the roles of expert and advocate relate to each other when parents are providing information on their child in different settings? As Goffman pointed out (1978), people present themselves differently in different settings according to the “rules of the game” and the intended outcome of the interaction. Parents, presenting themselves and their child as one unit (Stringer et al., 2018), may advocate on behalf of their child with the help of pictures and stories that seem to support their views in a specific situation (Graham et al., 2009).

In addition, parents as experts can, in spite of their expertise, also misread their child's intended communication, as has been described in literature focusing on video observations, in which outsiders saw communication signals that proxies missed (Daelman, 2003). A possible explanation for this discrepancy is the open mind of outsiders on the one hand, and the expectations and emotional involvement of parents that could cloud their interpretations on the other (Daelman, 2003).

That a parent can be seen as an advocate, who is working towards a favoured outcome for the situation of his/her child, and as an expert who can misread his/her child's signs, raises the question how other caregivers and medical professionals should position themselves, when listening to the stories of parents. Some authors (Olsman et al., submitted; Rosenbaum, King, Law, King, & Evans, 1998) have stressed that trust should be the basis for such interaction and that the listeners should refrain from making judgements based on a belief in an objective absolute truth, in order to leave space for the parents’ testimony. Olsman et al. (submitted) also argued that within such a relationship of trust, critical questions can be asked. This emphasis on trust is underscored by our findings, which suggest that parents have valuable knowledge of their child (Axelsson et al., 2014; Carter et al., 2017; Hostyn & Maes, 2009; Zaal‐Schuller, Willems, et al., 2016; de Geeter et al., 2002).) and that parents are more satisfied with the medical care for their child if their knowledge is valued as such by medical and other professionals (Stringer et al., 2018; de Geeter et al., 2002). At the same time, medical and other professionals have valuable expertise when it comes to assessing the situation of a person with PIMD (Daelman, 2003; Holenweg‐Gross et al., 2014; Takahashi & Tanaka, 2018). This being said, we should add that the knowledge of professionals is not objective knowledge either, especially in relation to this group.

Therefore, the understanding of, and the care for, people with PIMD can perhaps be best understood as a form of co‐production between the experience‐driven partly embodied knowledge of the parent and the knowledge of professionals. Such a complementary viewpoint can help to prevent polarization between parents and professionals (Stringer et al., 2018; Daelman, 2003; de Geeter et al., 2002; Rosenbaum et al., 1998) and can be of benefit in assessing complex multi‐interpretable situations such as the well‐being of a person who cannot communicate (Petry & Maes, 2006; Nieuwenhuijse, Willems, van Goudoever, Echteld, & Olsman, 2017; Olsman et al., submitted; Rosenbaum et al., 1998).

### 4.4 Clinical Implications

Parents’ embodied knowledge is regarded as important in providing good care for persons with PIMD, and therefore, parents should have the possibility to use this knowledge, and professionals should create space for the parents to do so (Graham et al., 2009; de Geeter et al., 2002). Through showing by example and by sharing stories and images, parents may help professionals acquire the knowledge they already possess of their child. Future research should further investigate this possibility of transferring embodied/tacit knowledge, including its practical feasibility and empirical effectiveness.

In addition, since it seems likely that professional caregivers can acquire the same type of knowledge as parents through emotional involvement and closeness, fuelled by interaction over time (Watson et al., 2017; Carter et al., 2016; Donovan, 2002; Reinders, 2010), professional caregivers should have a certain discretionary space to plan their own scheduling. In other words, the special and close relationship between professional caregivers and their clients with PIMD needs time and space to evolve. Therefore, care organizations should be wary of setting professional caregivers predetermined tasks, which they have to execute in predetermined amounts of time, and while doing so, deliver objectively measurable quality of care. Fulfilling these tasks could hamper professional caregivers in developing a special bond (Reinders, 2010). To allow such a bond to develop, furthermore, professional caregivers ideally should be given incentives (financial or otherwise) to work at a certain location and in a specific position for a longer period of time, thus counteracting the high turnover of personnel (Axelsson et al., 2014).

Our findings imply that the experiential knowledge about persons with PIMD is extremely fragile, since it is often the parents who fulfil an important role in their care (Gauthier‐Boudreault et al., 2017; Jokinen & Brown, 2005), sometimes for their entire lives (Seltzer et al., 2001), and this makes these parents the possessors of an in‐depth form of knowledge of their child. Although most people with PIMD live in professional support homes, some are known to live at home with their parents (Vlaskamp, 2002; Vugteveen et al., 2014). In this light, it might be useful if professional care organizations try to establish contact with ageing parents of these persons with PIMD, to start talking about the future care for their child (Brennan, Murphy, McCallion, & McCarron, 2018). This should be done in a delicate way, as families often find it hard to talk about the plans for the future (Heller & Kramer, 2009). In this regard, it is worth exploring the reasons for parents’ fear of being survived by their child with PIMD (Kamstra, Putten, & Vlaskamp, 2017; Luijkx & Vlaskamp, 2012). How does this fear relate to the prospect of their future absence as an expert and/or advocate of their child? What do parents see as possible solutions to fill the gaps that they might leave behind after they are gone? And which roles can siblings play in relation to this possible gap (Heller & Kramer, 2009; Luijkx, Putten, & Vlaskamp, 2016; Rawson, 2010)? These questions are clinically relevant and should be addressed in future studies.

## 5 CONCLUSION

This study has provided insight into the particular knowledge of parents and has shown how this knowledge can be used to improve the support and care for people with PIMD. Suggestions have been made for ways to retain and transfer this knowledge. We hope that this synthesis study is a first step not only towards providing a theoretical background for parents’ knowledge and how this knowledge is crucial in the understanding of and care for people with PIMD, but also in addressing the challenges ahead. These challenges include the arrangement of proper care for this vulnerable group within the context of imminent loss of knowledge.

## CONFLICT OF INTEREST

No potential conflict of interest was reported by the authors.

## Supporting information

Supplementary Material

Supplementary Material
