# Supplementary material for: Parents' knowledge of their child with profound intellectual and multiple disabilities: An interpretative synthesis
Source: J Appl Res Intellect Disabil. 2020 May 5;33(6):1141–50. doi: 10.1111/jar.12740 (PMC7687241; doi:10.1111/jar.12740)
Supplement: Supplementary file 1 — Supplementary Material [file JAR-33-1141-s001.docx]

**Appendix 1: Searches in databases**

17-1-2019

PubMed, PsycINFO, CINAHL, Philosophers’ Index

17 January 2019

| Databases: |  |  |
| --- | --- | --- |
| PubMed, PsycINFO (Ovid), CINAHL (Ebsco), Philosophers’ Index (Ebsco) | Before deduplication | After deduplication |
| Total | 2536 | 1939 |

Searches on 17 January 2019:

**PubMed**

1068 hits:

("Intellectual Disability"[Mesh] OR "Developmental Disabilities"[Mesh] OR profound cognitive impair*[tiab] OR multiple disabilit*[tiab] OR intellectual disabilit*[tiab] OR developmental disabilit*[tiab])

AND

(profound*[tiab] OR severe*[tiab])

AND

("Parents"[Mesh] OR "Caregivers"[Mesh] OR parent*[tiab] OR mother*[tiab] OR father*[tiab] OR family[tiab] OR families[tiab] OR relative*[tiab] OR caregiver*[tiab] OR relation*[tiab] OR partner*[tiab])

AND

("Health Knowledge, Attitudes, Practice"[Mesh] OR knowledge*[tiab] OR knowing[tiab] OR feel*[tiab] OR sense[tiab] OR understand*[tiab] OR information*[tiab] OR experien*[tiab] OR tacit*[tiab])

**PyscINFO (Ovid)**

967 hits:

| # | Searches | Results |
| --- | --- | --- |
| 1 | exp intellectual development disorder/ or exp multiple disabilities/ or exp developmental disabilities/ or (profound cognitive impair* or multiple disabilit* or intellectual disabilit* or developmental disabilit*).ti,ab,id. | 62615 |
| 2 | (profound* or severe*).ti,ab,id. | 152675 |
| 3 | exp parents/ or caregivers/ or (parent* or mother* or father* or family or families or relative* or caregiver* or relation* or partner*).ti,ab,id. | 1565920 |
| 4 | health knowledge/ or knowledge level/ or health attitudes/ or (knowledge* or knowing or feel* or sense or understand* or information* or experien* or tacit*).ti,ab,id. | 1566621 |
| 5 | 1 and 2 and 3 and 4 | 967 |

**CINAHL (Ebsco)**

452 hits:

(MH "Intellectual Disability+") OR (MH "Developmental Disabilities") OR ( TI ( profound cognitive impair* or multiple disabilit* or intellectual disabilit* or developmental disabilit* ) OR AB ( profound cognitive impair* or multiple disabilit* or intellectual disabilit* or developmental disabilit* ) )

AND

TI ( profound* OR severe* ) OR AB ( profound* OR severe* )

AND

(MH "Parents+") OR (MH "Caregivers") OR ( TI ( parent* or mother* or father* or family or families or relative* or caregiver* or relation* or partner* ) OR AB ( parent* or mother* or father* or family or families or relative* or caregiver* or relation* or partner* ) )

AND

(MH "Knowledge+") OR (MH "Attitude to Health+") OR ( TI ( knowledge* or knowing or feel* or sense or understand* or information* or experien* or tacit* ) OR AB ( knowledge* or knowing or feel* or sense or understand* or information* or experien* or tacit* ) )

**Philosophers Index (Ebsco)**

49 hits:

DE "DISABILITY"  OR DE "DEVELOPMENTAL DISABILITY" OR DE "COGNITIVE DISABILITY" OR TI (cognitive impair* or multiple disabilit* or intellectual disabilit* or developmental disabilit* ) OR AB ( cognitive impair* or multiple disabilit* or intellectual disabilit* or developmental disabilit* )

AND

TI ( profound* or severe* ) OR AB ( profound* or severe* )

AND

DE "PARENT" OR DE "FAMILY" OR DE "MOTHERHOOD"  OR DE "PARTICIPATION" OR TI ( parent* or mother* or father* or family or families or relative* or caregiver* or relation* or partner* ) OR AB ( parent* or mother* or father* or family or families or relative* or caregiver* or relation* or partner* )
