# Supplementary material for: Parents' knowledge of their child with profound intellectual and multiple disabilities: An interpretative synthesis
Source: J Appl Res Intellect Disabil. 2020 May 5;33(6):1141–50. doi: 10.1111/jar.12740 (PMC7687241; doi:10.1111/jar.12740)
Supplement: Supplementary file 2 — Supplementary Material [file JAR-33-1141-s002.docx]

**Appendix 2: Thematic analysis of the literature, schematically presented**

| **1st author** | **1. What is the nature of parents’ knowledge of their child with PIMD?** | **2. How do they use this knowledge?** | **3. Is this knowledge transferable to others?** |
| --- | --- | --- | --- |
| Axelsson | - Expressions of a child with PIMD are not always perceived as easily interpretable.  - Successful communication requires high degrees of knowledge and experience | As an expert, to accomplish successful participation (in activities), which seemed to depend on understanding and interpreting the child’s emotions and wishes. | - |
| Carter | - Simply ‘knowing’ - Instinctive and intuitive knowledge  - Learned over the years.  - Guessing / working out. | As both an expert and an advocate regarding the assessment of pain. | - |
| Carter | - A sense of knowing.  - A gut feeling. | As both an expert and an advocate regarding the assessment of pain. | - |
| Fonteine | - Rich and in-depth experiential knowledge. | - | Not transferable through logs at activity centers.  Try to transfer in-depth knowledge by studying interactions between parent and child |
| Gauthier-Boudreault | - | As an advocate to ensure their child receives good care during his/her life course. | Fear of own ageing in relation to the loss of knowledge about the child and the advocacy on behalf of the child. |
| Gauthier-Boudreault | - Knowing the child is important for providing good care. | - | - |
| Geeter | - Parents can provide reliable and valuable information on their child.  - Based on their special bond and long-standing experience with their child. | As an expert, providing information to, and co-producing with, schools to improve their child’s education. | A parent needs space in a professional organization to make use of his/her expertise, e.g. a formal position within the professional organization. |
| Graham | - A lack of appreciation (among PICU providers) of the abilities of the children and their level of functioning was strikingly evident to parents. | As an advocate providing information on the child to influence the views of medical professionals. | In a PICU, parents should routinely be asked what their child is like at home. |
| Hostyn | - Professionals may lack connectedness with and responsivity to the clients' capacities and needs.  - This may result in low quality interaction. | Parents (and other emotionally involved persons) functioned as experts in achieving successful interaction with their child with PIMD. | Disability support workers indicated the importance of getting to know the client by spending time together. Parents and professionals stated that successful interaction is characterized, among other factors, by emotional attachment. |
| Hunt | - Just knowing.  - Comparing with the ‘normal situation’.  - A feeling / An intuition.  - Something that comes from within you. | As both an expert and advocate regarding the assessment of pain. | Knowing the child’s parents can bring the  professional closer to knowing the child as a person.  Nurses (…) got to know the patient through the patient’s family. They used the  family’s ability to recognize minute changes in the  patient and what would comfort and discomfort the  patient. |
| Stringer | - Best equipped, due to their close bond, to understand the attempts at communication of the persons with PIMD. | As an advocate in representing their child with PIMD in relation to the care provided by the family physician. | - |
| Watson | - Knowledge of the personal history of the person with PIMD is important for feeling relational closeness  - Relational closeness is a key factor in responsiveness. | Parents (and other emotionally involved persons) functioned as experts in understanding the communication and preferences of persons with PIMD. | The sharing of life stories, images and videos of the person being supported, by those who have known them for a long time, across multiple areas of their lives, was found to enhance other supporters’ knowledge of the person. |
| Zaal-Schuller | - A sense of their child's condition and needs.  - An intuition. | As an expert regarding end-of-life decisions for their child. | - |
| Zaal-Schuller | - ‘Reading’ the child.  - Feeling when their child was deteriorating before the physician was aware. | As both an expert and advocate on QoL and wellbeing of their child, in relation to end-of-life decisions regarding their child | - |
